# Impact of transmitted CTL escape mutations on replicative capacity and HIV pathogenesis in early infection

**DOI:** 10.1186/1742-4690-9-S2-O57

**Published:** 2012-09-13

**Authors:** J Prince, D Claiborne, D Heckerman, J Carlson, H Prentice, M Schaefer, L Yue, J Mulenga, J Tang, P Goepfert, P Farmer, R Kaslow, S Allen, E Hunter

**Affiliations:** 1 Emory University, Atlanta, GA, USA; 2 Microsoft Research, Los Angeles, CA, USA; 3 University of Alabama Birmingham, Birmingham, AL, USA; 4 Zambia Emory HIV Research Project (ZHERP), Lusaka, Zambia

## Background

Multiple HLA class I alleles have been shown to influence both HIV-1 transmission and viral load. In transmission pairs, viral loads of acutely infected partners correlate with viral loads (VL) of their chronically infected donors. This correlation becomes highly significant after adjustment for host factors known to modulate viral load. In addition, we have previously demonstrated that transmission of a virus containing multiple HLA-I associated polymorphisms resulted in a lower set point VL in Zambian linked recipients. These studies imply that transmitted viral characteristics play a role in defining early HIV-1 pathogenesis, and it will be important for vaccine development to understand which viral characteristics are responsible for this.

## Methods

We investigated the role that the in vitro replicative capacity (RC) of the transmitted Gag plays in defining HIV-1 clinical parameters, by cloning gag genes from the founder virus in newly infected partners of 149 epidemiologically linked transmission pairs into the subtype C, R5 tropic MJ4 provirus.

## Results

We observed a statistically significant positive correlation between the RC of Gag-MJ4 chimeras and set point VL in seroconverters (P=0.013). The RC of the transmitted Gag also correlated (P=0.025) to the viral load in the chronically infected donor, pointing to RC as the major viral characteristic responsible for the link between donor and linked recipient viral loads. The long term clinical benefit associated with the transmission of attenuated viruses was investigated by performing a Kaplan Meier analysis of time to CD4+ count less than 350. Individuals infected with attenuated gag sequences (RC< 1) were delayed in their progression to CD4+ counts < 350 compared to high (RC >2) replicating viruses (P = 0.034).

## Conclusion

Interestingly, this phenomenon seemed to be independent of viral load perhaps highlighting the role that early viral replication may play in defining HIV-1 pathogenesis.

